# Infant expectations of instant or delayed gratification

**DOI:** 10.1038/s41598-020-76136-9

**Published:** 2020-11-05

**Authors:** Yuyan Luo, Duangporn Pattanakul

**Affiliations:** Department of Psychological Sciences, University of Missouri, Columbia, MO USA

**Keywords:** Evolution, Psychology

## Abstract

Choices between immediate gratification and long-term (but larger) gains are prevalent in human life, which is why the decision-making processes to delay gratification have been studied extensively throughout different developmental ages. Children’s delay-of-gratification behaviors have been examined in the well-known “marshmallow test,” in which 3- to 5-year-olds are given a marshmallow and told by an experimenter that they can eat it immediately or wait for an unspecified duration of time (which can be capped at 15 min) until the experimenter returns so that they can receive another marshmallow. Children's wait time has been viewed as a good indicator of their later development. Here we show that a group of 22-month-old infants (*N* = 32) already held expectations about others’ choices in a violation-of-expectation looking-time task modeled after the marshmallow test. The infants expected an agent to defer gratification based on a speaker’s promise of the second marshmallow available in the future, but to eat the currently attainable marshmallow when the speaker made no such promise. Our findings indicate an early-emerging understanding of others’ choices of delayed or instant gratification and shed new light on the development of delay-of-gratification behaviors.

## Introduction

People routinely make decisions on instant or deferred gratification, especially in situations concerning food intake. This is exactly the challenge faced by preschoolers in the delay-of-gratification paradigm^1^. Various factors sway children’s decision of eating or waiting in the marshmallow test, for instance, their self-control skills—the ability to resist the temptation of instant reward (e.g., by distracting themselves) in order to obtain larger gains at a later point in time^1,2^, and children’s trust in the experimenter’s promise of the second marshmallow^3–6^. Importantly, children's wait time in the marshmallow test is considered a good predictor of their later development^7–9^ (see^10–12^ for recent conversations on how social and environmental factors influence these longitudinal links). It remains unexplored, however, whether some early understanding about delay-of-gratification exists before individual differences can be observed in preschool years. That is, do infants hold general expectations about others’ choices of delayed or instant gratification, before children are old enough to participate in the marshmallow test? The present study addressed this question.

There have been numerous reports of infants’ understanding about the physical, psychological, and even moral domains of the world from the violation-of-expectation looking-time tasks^13–15^. The study rationale is that infants typically respond with heightened interest or prolonged looking to events that are inconsistent, as opposed to consistent, with their expectations. By measuring looking responses, these studies alleviate the requirements on the part of the young child to act on their knowledge (e.g., to answer an experimenter’s questions) and thus yield findings that suggest the early emergence of cognitive competencies. The present study took a similar approach. Instead of asking children to wait patiently in the marshmallow test, we tested infants in a violation-of-expectation looking-time task modeled after the marshmallow test to gauge their early understanding.

**Figure 1:**
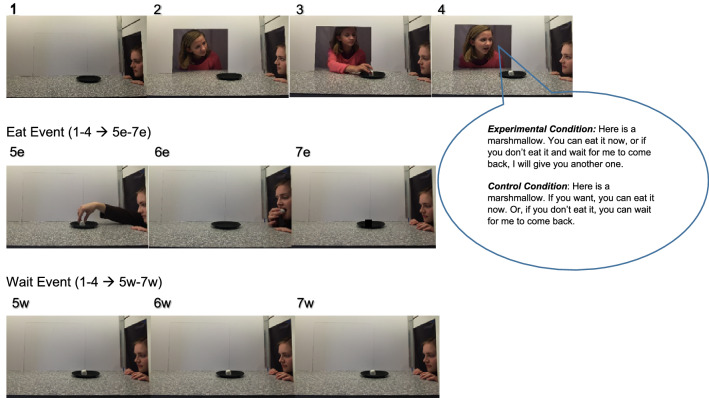
Schematic depiction of the two types of events shown to the infants in the experimental and control conditions. In each event, after watching the scene with an agent and an empty plate in front of her (1), the action sequence began in which a speaker appeared by opening a window, put a marshmallow on the plate, talked to the agent, and then left the apparatus by closing the window (2–4). The two conditions only differed in what the speaker said to the agent. In the eat event, the agent ate the marshmallow (5e–6e) and then tapped her fingers on the apparatus in the main trial (7e) until the trial ended. In the wait event, the agent never touched the marshmallow and only tapped her fingers (5w-6w). She kept tapping her fingers in the main trial (7w) until the trial ended.

In the study, full-term, monolingual English-learning infants (*N* = 32) were randomly assigned to an experimental or a control condition, 17 male (age range: 18 months, 26 days to 24 months, 18 days; *M* = 21 months, 25 days). We chose this age group based on previous infant studies with similar designs^16,17^. In the *experimental* condition, infants received six trials alternating between two types of events, wait or eat event, that involved two experimenters, a speaker and an agent (see Fig. 1). In both events, to start, the agent sat by a side window of an apparatus that resembled a stage, with an empty plate in front of her. Next, the speaker appeared by opening a window on the back of the stage. She looked at the agent, placed a marshmallow on the plate, and said to the agent, in infant-directed speech^18^, “Here is a marshmallow. You can eat it now, or if you don’t eat it and wait for me to come back, I will give you another one.” The speaker then exited the apparatus by closing the back window. The agent either waited while tapping her fingers on the apparatus (wait event) or ate the marshmallow and then tapped her fingers (eat event). The *control* condition was identical to the experimental condition except for what the speaker said to the agent. Instead of making a promise of an additional marshmallow, she simply said, “Here is a marshmallow. If you want, you can eat it now. Or, if you don’t eat it, you can wait for me to come back.” The sentences used in the two conditions were similar in structure as those used with infants at similar ages^17^.

Previous research has shown that toddlers are trusting of what other people say, even when it conflicts with their first-hand experiences^19–21^. This default to trust others’ testimony is viewed as adaptive and serves cognitive and social functions early in life, e.g., to lay the foundations of language acquisition, or to establish connections with others^22–24^. Therefore, in the present study, infants would likely trust what the speaker said. In the experimental condition, if infants expected the agent to wait based on what the speaker had said to her, then they should respond with heightened interest when the agent ate the marshmallow immediately. We predicted that infants would look reliably longer at the eat event than at the wait event. In the control condition, by contrast, the speaker only listed to the agent the two choices, to eat or to wait, in the same order as in the experimental condition. There was no promise of a second marshmallow as the result of waiting. In this condition, infants should expect the agent to act on instant gratification and eat the marshmallow, as we humans have evolved to meet our basic needs for food^25^. We predicted that infants in the control condition would look reliably longer at the wait event than at the eat event. Thus, we hypothesized to obtain opposite patterns of results from the two conditions, although the two conditions were highly similar. Note that in the present study, the role of the agent was to carry out the actions of eating or waiting so that infants’ looking responses to these actions could be measured. There was no requirement for infants to make any inferences about what the agent expected and/or the interaction or relationship between the agent and the speaker.

## Results

Infants received six trials alternating between the eat and the wait event, appropriate for their condition. Each trial consisted of a fixed-length action sequence (1–6 in Fig. 1) and a main trial (7 in Fig. 1). Infants’ looking times in the main trial of the six trials were log-transformed to reduce positive skewness^26^. Analyses were performed on log looking times; raw looking times are provided below to facilitate communication.

**Figure 2:**
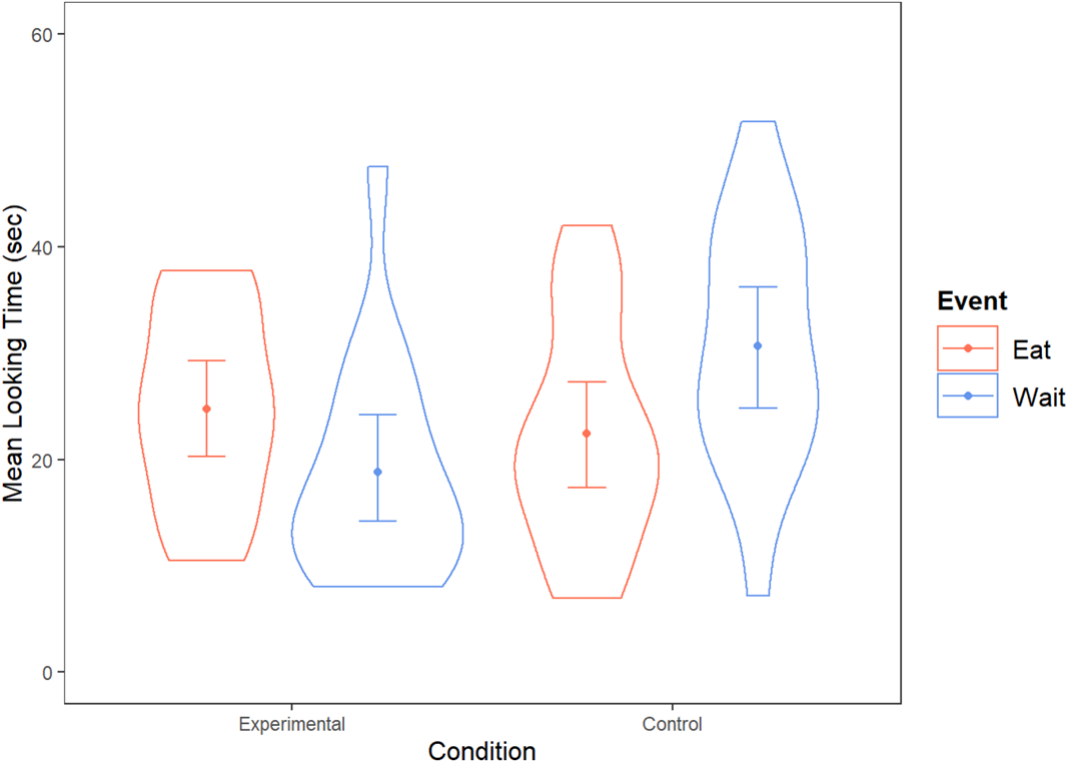
Violin plot of infants’ mean looking times at the two types of events (eat or wait) in the experimental and control conditions. Dots represent condition means and error bars represent 95% confidence intervals. The kernel density plot on each side of the lines shows the probability of the data at different proportions.

Infants’ log main-trial looking times were averaged and analyzed using a 2 × 2 repeated-measure analysis of variance (ANOVA) with condition (experimental or control) as a between-subjects factor and event (wait or eat) as a within-subject factor. The analysis yielded a significant Condition x Event interaction, *F*(1, 30) = 15.83, *p* = 0.0004, *η*_*p*_^2^ = 0.345. No other effect was significant. Planned comparisons revealed that infants in the experimental condition looked reliably longer at the eat event (*M* = 24.74 s, *SD* = 9.15) than at the wait event (*M* = 18.79 s, *SD* = 10.45), *F*(1, 30) = 7.83, *p* = 0.009, Cohen’s *d* = 0.657, *t*(15) = 2.68, Scaled Jeffreys-Zellner-Siow (JZS) Bayes Factor = 3.521^27^ in favor of the alternative hypothesis that infants would look differently at the two events over the null hypothesis, while those in the control condition looked reliably longer at the wait event (*M* = 30.67 s, *SD* = 11.49) than at the eat event (*M* = 22.42 s, *SD* = 10.64), *F*(1, 30) = 8.03, *p* = 0.008, Cohen’s *d* = 0.705, *t*(15) = 2.96, Scaled JZS Bayes Factor = 5.593 in favor of the alternative hypothesis (see Fig. 2). Nonparametric Wilcoxon signed-ranks tests confirmed these results (Experimental condition: *z* = 2.17, two-tailed *p* = 0.03; Control condition: *z* = 2.53, two-tailed *p* = 0.011).

In addition, we ran three analyses of covariance (ANCOVA) to examine the effect of infants’ age, comprehensive or productive vocabulary size (as measured by the MacArthur Communicative Development Inventories (MCDIs) filled out by parents^28^), on the main results. With age (in days) as the covariate, the results replicated those of the ANOVA: the Condition x Event interaction was significant, *F*(1, 29) = 11.78, *p* = 0.0018. Planned comparisons confirmed that infants in the experimental condition looked reliably longer at the eat than at the wait event, *F*(1, 29) = 6.44, *p* = 0.017, whereas those in the control condition did the reverse, *F*(1, 29) = 6.64, *p* = 0.015. These results still held when infants’ comprehensive or productive vocabulary size was each included as the covariate (comprehensive vocabulary size: Condition x Event interaction: *F*(1, 29) = 15.13, *p* = 0.0005; differences in the experimental condition: *F*(1, 29) = 7.52, *p* = 0.010; differences in the control condition: *F*(1, 29) = 7.74, *p* = 0.009; productive vocabulary size: Condition x Event interaction: *F*(1, 29) = 15.28, *p* = 0.0005; differences in the experimental condition: *F*(1, 29) = 7.54, *p* = 0.010; differences in the control condition: *F*(1, 29) = 7.75, *p* = 0.009).

### Additional analyses

The results above were obtained with infants’ log looking times averaged across three pairs of trials. To further examine infants’ responses throughout the experiment, we first ran an ANOVA with condition as a between-subjects factor and event and trial pair (first, second, or third) as within-subject factors, although some infants did not contribute data to all three pairs (see Methods). While the Condition x Event interaction remained significant (*F*(1, 134) = 9.75, *p* = 0.002), there was no significant interaction of Condition x Event x Trial pair (*F*(2, 134) = 2.20, *p* = 0.115), suggesting that the effects in the two conditions did not change significantly across the three pairs of trials. Next, we performed paired *t*-tests on infants’ log looking times at the two types of events in the first, second, and third pair of trials within each condition. In the experimental condition, infants looked longer at the eat event than at the wait event in all three pairs (see Table 1). The difference was significant in the first pair, *t*(15) = 3.60, two-tailed *p* = 0.003, but not in the second (*t*(14) = 0.35, two-tailed *p* > 0.250) or the third pair (*t*(12) = 1.07, two-tailed *p* > 0.250). In the control condition, infants looked longer at the wait than at the eat event in the first and third pairs (see Table 1). Similar to the experimental condition, the difference was only significant in the first pair, *t*(15) = 2.92, two-tailed *p* = 0.011 (second pair: *t*(14) = 0.06, two-tailed *p* > 0.250; third pair: *t*(12) = 1.57, two-tailed *p* = 0.143). Therefore, in both conditions, while the respective effect was significant only in the first pair of trials, the effects did not vary by trial pair and the numerical differences in infants’ looking time at the two events emerged even in the third pair of trials.

**Table 1:** Mean looking times (in seconds) and standard deviations (in parentheses) at the two types of events in the first, second, and third pair of trials.

	Eat event	Wait event
**Experimental condition**		
First pair	28.23 (13.88)*	18.39 (13.45)
Second pair	25.31 (17.91)	23.31 (16.54)
Third pair	20.01 (15.35)	13.97 (8.16)
**Control condition**		
First pair	30.19 (23.20)	47.86 (27.95)*
Second pair	24.90 (20.37)	23.97 (13.99)
Third pair	12.15 (6.30)	22.08 (22.75)

*Greater than the other event, two-tailed *p* < .05.

## Discussion

These results suggest that while watching other people act, infants seem to hold general expectations of others’ choices between delayed or instant gratification. In the violation-of-expectation task modeled after the marshmallow test^1^, when the speaker put a marshmallow in front of the agent and also promised her a second marshmallow if she waited in the experimental condition, infants seemed to have expected the agent to wait and responded with heightened interest when the agent ate the marshmallow. This expectation persisted across three pairs of trials, even though the speaker never fulfilled her promise throughout the experiment. Therefore, consistent with prior suggestions of an early default to trust what others say^21^, the infants appeared to believe in what the speaker said to the agent and made predictions about the agent’s actions accordingly. In the control condition, in contrast, the speaker did not make a promise to the agent, infants hence seemed to expect the agent to eat the marshmallow and responded with heightened interest when she waited instead.

Therefore, even before their second birthday, infants expect others to wait and not eat an available marshmallow if given a promise of a second one. How do infants come to this appreciation? We speculate that infants’ own experiences might contribute to the understanding, in addition to their trust in others’ words. In the first two years of life, infants become increasingly adept at self-regulation, that is, to regulate and monitor their own behaviors and emotions in accord with external requirements and demands, for example, to comply with caregivers’ requests and eventually internalize these requests^29–31^. In a situation in which an infant’s mother told him/her *not* to touch attractive toys that were easily accessible for eight minutes, for instance, the compliance rate gradually improved as infants developed, from 40% at 14 months to 78% at 22 months^32^. There are noticeable similarities between this situation and the present study, e.g., the requested inhibition of a powerful response of touching attractive toys or eating a marshmallow. It is thus plausible that while infants themselves are tasked with learning what is expected of them and complying with requests and even prohibitions from adults, they may also formulate a general expectation of compliance. In the present study, this expectation would have been satisfied by either one of the agent’s choices, to eat, or not to eat but wait. In the experimental condition, however, infants’ trust in the speaker’s promise of a reward might have led to their predication that the agent should wait and not eat the marshmallow immediately. On the other hand, in the control condition, without this promise, the desire of consuming the marshmallow might have taken precedence and led to the prediction that the agent should eat.

The fact that the effects were found in the present study suggests that infants might have understood the gist of the speaker’s speech, although we had no direct evidence for it. In our view, the important difference in the speaker’s speech between the two conditions was the words “give,” “and,” and “another” (e.g., as in the speaker’s promise “*and … I will give you another one*” in the experimental condition). There is indirect support for infants’ comprehension of these words. For example, compiled CDI data^33^ show that most 18-month-old infants can understand “give” and by 22 months, about 20% of infants can even *produce* “and” and “another.” A recent study^34^ further shows that 14- to 18-month-old Korean infants formed different expectations about an object’s (i.e., a ball) location when they heard “the ball *and* the cup” versus “the ball is in the cup,” pointing to early comprehension. However, in the present study, the speaker also used conditionals (the “*if*” sentences) in both conditions, which infants in the current age range rarely *produce*^33,35,36^*.* To our knowledge, no study has directly examined young children’s understanding of conditionals. Nevertheless, recent work^37–40^ on infants’ and toddlers’ logical reasoning (A or B, if not A, then B) based on visual or linguistic information can speak to the possibility that rudimentary cognitive structures might be in place early in life to enable comprehension of conditionals. Additionally, conditionals exist in parental input even to their 12-month-olds, at least in structured caregiver-infant interactions^41^. This again seems consistent with our speculation that the effects found in the present study, which are unrelated to infants’ vocabulary size, despite the variability of their age, may stem from these infants’ socialization experiences.

The present study provides experimental evidence that infants hold expectations about instant or delayed gratification. It is still an open question how these early expectations translate to what infants do themselves (e.g., whether or how to embrace parental demands) and later preschoolers’ responses in the delay-of-gratification paradigm (e.g., whether or how long to wait for an experimenter to get more treats). As mentioned earlier, children’s self-control skills affect their delay-of-gratification behavior. Self-control is a broad construct that includes not only cognitive skills of executive functions (e.g., working memory, inhibitory control), but also self-regulation acquired through socialization^42^. While variances in cognitive skills, which are also shaped by experiences such as socialization^43^, can contribute to the individual differences found in the delay of gratification, the importance of the social and environmental influences on children’s self-control has been highlighted by recent work^44–48^. For example, children may delay gratification because of cultural and/or parental expectations, or to conform to their in-group behavior^45,47^. Similarly, with regard to the development of self-regulation, researchers have also taken an integrative approach to examine a variety of social and environmental factors such as children’s temperaments, parent–child relationships, and parenting practices, in addition to executive functioning skills^30,49–51^. In the present study, these factors were not considered. It is possible that the expectations of delayed gratification found in the present study are held by most if not all infants, despite their individual differences, presumably because of the general expectation of compliance and the default to trust others’ words. It is also possible that there already might be differences in infant expectations because of variances in a wide range of factors such as caregivers’ demands on infants, parent-infant interactions, and infants’ experiences inside and outside their home environment. For instance, in resource-limited environment, delaying may not be adaptable, or individuals may be reluctant to trust others’ promises because of a lack of reliability or predictability they have encountered^3,48^. This might lead to infants holding expectations different than those found in the present sample (e.g., they might expect the agent to ignore the speaker’s promise and eat the marshmallow in the experimental condition). Therefore, uncovering infant understanding before children can wait patiently themselves, e.g., in the marshmallow test, can help to elucidate the cognitive, social, and environmental influences on the dynamic development of delay-of-gratification behaviors.

## Methods

### Participants

Thirty-two healthy, full-term, monolingual English-learning infants, 17 male, participated (range: 18 months, 26 days to 24 months, 18 days; *M* = 21 months, 25 days). Sixteen infants, 8 male, were randomly assigned to the experimental condition (*M* = 21 months, 14 days, *SD* = 36 days), and the rest to the control condition (*M* = 22 months, 7 days, *SD* = 35 days). This sample size was determined by a power analysis using an effect size *f* of 0.25 (Cohen’s *d* is approximately 0.5) and an alpha level of 0.05 for the *F*-test of a 2 × 2 between-within interaction design, which can achieve power of at least 0.75^52^. Another four infants were tested but excluded because of differences in test looking times more than 3 *SD*s from the mean of the condition (*n* = 2), being distracted (*n* = 1), or being exposed regularly (~ 90%) to a language other than English based on parental input (*n* = 1). Data collection for the present study was stopped after these 36 infants participated. Of the 36 infants, 86.11% were White, 5.56% Asian, 5.56% multiracial, and 2.78% African American. The study was approved by the University of Missouri Institutional Review Board. The methods were carried out in accordance with the approved guidelines. Informed written consent was obtained from a parent or legal guardian of the participating infants. We also obtained informed written consent from the two experimenters shown in Fig. 1 of the study participation and publication of their images in an online open-access journal.

### Apparatus

The apparatus consisted of a wooden display box (106 cm high × 104 cm wide × 61 cm deep) mounted 76 cm above the room floor. The infant sat on a parent’s lap and faced an opening (56 cm high × 102 cm wide) in the front of the apparatus. Between trials, a curtain consisting of a muslin-covered frame (61 cm high × 104 cm wide) was lowered in front of the opening. The side walls of the apparatus were painted white, and the floor was covered with a foam board wrapped with grey granite patterned contact paper. A rectangular window (31.5 cm high × 30.5 cm wide) was created in the right-side wall. The agent, wearing a brown shirt, sat by this window during the experiment. The speaker, wearing a pink shirt, sat behind a rectangular window (35.5 cm high × 44.5 cm wide) created in the mid-section of the back wall made of white foam board. A large white cloth curtain covered the area behind her.

A round paper plate, 17 cm in diameter, was painted black and used in the experiment. On the apparatus floor, the plate was 21.5 cm from the agent’s window and 7 cm from the back wall. Kraft Jet-Puffed white marshmallows were used. Each cylindrical marshmallow measured approximately 2.5 cm in diameter and 3 cm high.

The apparatus was also equipped with two video cameras. One recorded the events being shown on the apparatus, while the other recorded the infant. The input from the two cameras could be monitored online and checked offline to ensure proper testing. A metronome that beat softly once per second was used to help the experimenters adhere to the scripts.

### Procedure

To determine an infant’s vocabulary, the parent(s) filled out the MacArthur Communicative Development Inventories (MCDIs)^28^ before the experiment began. MCDIs are widely used parental reports for assessing communicative skills in infants and toddlers. The Level II short form MCDIs (Form A, for 16- to 30-month-olds) with 100 words listed were used. Parents checked how many of them their infant could comprehend and/or produce.

During the experiment, the infant sat on the parent’s lap in front of the apparatus. Parents were instructed to close their eyes during the trials and not interact with the infants. After being seated in front of the apparatus, infants were greeted by the two experimenters, the speaker and the agent, one at a time. The speaker was always a native speaker of English. The experimenters were blind to the hypotheses of the study and were also required to follow the scripts (see below) designed to ensure that the events were presented similarly to each infant. Two naïve observers monitored the infant's looking behavior by viewing the infant through peepholes in large cloth-covered frames on either side of the apparatus. Each observer held a controller linked to a computer software^53^ and pressed the button when the infant looked at the event. Looking times recorded by the primary observer were used for data analyses. For 8 of the 32 infants, only the primary observer was present. Inter-observer agreement for the remaining 24 infants averaged 93% per trial per infant.

In the experimental condition, the infants received six trials alternating between the eat and the wait events. Each event consisted of a 38-s action sequence and a main trial; looking times were computed separately. To start, the infant looked at the scene with the agent and the empty plate in front of her for 2 cumulative seconds. Next, the action sequence began. The speaker opened the back window (5 s). The agent and the speaker turned to look at each other (2 s). The speaker then put a marshmallow on the plate (3 s) and said to the agent, “Here is a marshmallow. You can eat it now, or if you don’t eat it and wait for me to come back, I will give you another one.” (13 s) The speaker exited the apparatus by closing the back window (5 s). Following this, the last 10-s segment of the action sequence differed between the two types of events. In the eat event, the agent grabbed the marshmallow and ate it (10 s). In the wait event, the agent tapped her fingers on the edge of the apparatus (10 s). In the main trial, the agent kept tapping her fingers, with the empty plate (eat event) or the plate with the marshmallow on it (wait event) in front of her. The main trial ended when infants looked away for 2 consecutive seconds after having looked for at least 5 cumulative seconds or looked for 90 cumulative seconds. The control condition was similar to the experimental condition except for what the speaker said to the agent after putting the marshmallow on the plate. Across the two conditions, 15 infants, 8 male, saw the eat event first, and the remainder saw the wait event first.

Infants were attentive during the 38-s action sequence of the trials (range: 30 to 38 s; experimental condition: *M* = 37.45 s, *SD* = 0.94; control condition: *M* = 36.69 s, *SD* = 2.01). Across the two conditions, 26 infants contributed data from all three pairs of trials. The remaining infants contributed data from the first pair (*n* = 2) or from the first two pairs of trials (*n* = 4) because of experimenter error, refusal to continue, observer difficulty, equipment malfunction, parental interference, or being distracted. For these infants, the last two or the last one pair of trials were treated as missing data.

The infants’ *comprehensive* vocabularies, as assessed by MCDI, were similar between the two conditions (experimental condition: range = 23 to 100, *median* = 70; control condition: range = 8 to 99, *median* = 64). The infants’ *productive* vocabularies, however, differed between the two conditions, perhaps due to the fact that the mean age of the infants in the control condition was 23 days older than that of the experimental condition (experimental condition: range = 5 to 96, *median* = 25; control condition: range = 3 to 70, *median* = 34.5). The Pearson Correlations tests nevertheless confirmed that these two measures of infant vocabulary size were positively correlated within each condition (experimental condition: *r*(14) = 0.709, two-tailed *p* = 0.002; control condition: *r*(14) = 0.758, two-tailed *p* = 0.001).

Preliminary analyses of the log mean looking time data revealed no significant interactions of condition and event with sex or order, all *F*s(1, 24) < 3.70, *p*s > 0.066; the data were therefore collapsed across sex and order in the main analyses.
